# An artificial intelligence prediction model outperforms conventional guidelines in predicting lymph node metastasis of T1 colorectal cancer

**DOI:** 10.3389/fonc.2023.1229998

**Published:** 2023-10-24

**Authors:** Zheng Hua Piao, Rong Ge, Lu Lu

**Affiliations:** Department of Pathology, Ningbo Clinical Pathology Diagnosis Center, Ningbo, Zhejiang, China

**Keywords:** T1 colorectal cancer, endoscopic resection, additional surgery, lymph node metastasis, artificial intelligence model

## Abstract

**Background:**

According to guidelines, a lot of patients with T1 colorectal cancers (CRCs) undergo additional surgery with lymph node dissection after being treated by endoscopic resection (ER) despite the low incidence of lymph node metastasis (LNM).

**Aim:**

The aim of this study was to develop an artificial intelligence (AI) model to more effectively identify T1 CRCs at risk for LNM and reduce the rate of unnecessary additional surgery.

**Methods:**

We retrospectively analyzed 651 patients with T1 CRCs. The patient cohort was randomly divided into a training set (546 patients) and a test set (105 patients) (ratio 5:1), and a classification and regression tree (CART) algorithm was trained on the training set to develop a predictive AI model for LNM. The model used 12 clinicopathological factors to predict positivity or negativity for LNM. To compare the performance of the AI model with the conventional guidelines, the test set was evaluated according to the Japanese Society for Cancer of the Colon and Rectum (JSCCR) and National Comprehensive Cancer Network (NCCN) guidelines. Finally, we tested the performance of the AI model using the test set and compared it with the JSCCR and NCCN guidelines.

**Results:**

The AI model had better predictive performance (AUC=0.960) than the JSCCR (AUC=0.588) and NCCN guidelines (AUC=0.850). The specificity (85.8% vs. 17.5%, *p*<0.001), balanced accuracy (92.9% vs. 58.7%, *p*=0.001), and the positive predictive value (36.3% vs. 9.0%, *p*=0.001) of the AI model were significantly better than those of the JSCCR guidelines and reduced the percentage of the high-risk group for LNM from 83.8% (JSCCR) to 20.9%. The specificity of the AI model was higher than that of the NCCN guidelines (85.8% vs. 82.4%, p=0.557), but there was no significant difference between the two. The sensitivity of the NCCN guidelines was lower than that of our AI model (87.5% vs. 100%, p=0.301), and according to the NCCN guidelines, 1.2% of the 105 test set patients had missed diagnoses.

**Conclusion:**

The AI model has better performance than conventional guidelines for predicting LNM in T1 CRCs and therefore could significantly reduce unnecessary additional surgery.

## Introduction

T1 colorectal cancer (T1 CRCs) with superficial submucosal invasion can be cured by endoscopic resection (ER); therefore, effective screening for LNM is very important. High-grade histology, lymphovascular infiltration (LV), depth of submucosal invasion (DSI) ≥1000 μm, and tumor budding are considered risk factors for lymph node metastasis (LNM) in most guidelines (1–4). According to those guidelines, approximately 57% to 91% of patients with T1 CRCs are classified in a high-risk group for LNM, while the rate of LNM is only 3.3% to 4.7% (5–8); thus, effective screening of high-risk patients will reduce the large number of patients who receive unnecessary surgery (5, 8).

According to guidelines, patients with any one histologic risk factor in the pathologic examination will be classified into the high-risk group for LNM and be recommended for additional surgery (1–3). However, even those patients who are classified into the high-risk group have varying degrees of LNM risk, as each patient has different types and combinations of risk factors (8–12). Therefore, it is necessary to establish a more effective method to predict the probability of LNM and choose the best treatment plan based on the degree of risk. Artificial intelligence (AI) models can simultaneously consider multiple risk factors and ultimately predict the risk of LNM. In this study, we aimed to develop an AI prediction model to more accurately identify patients with a high risk of LNM.

## Methods

### Study cohort

**Figure 1:**
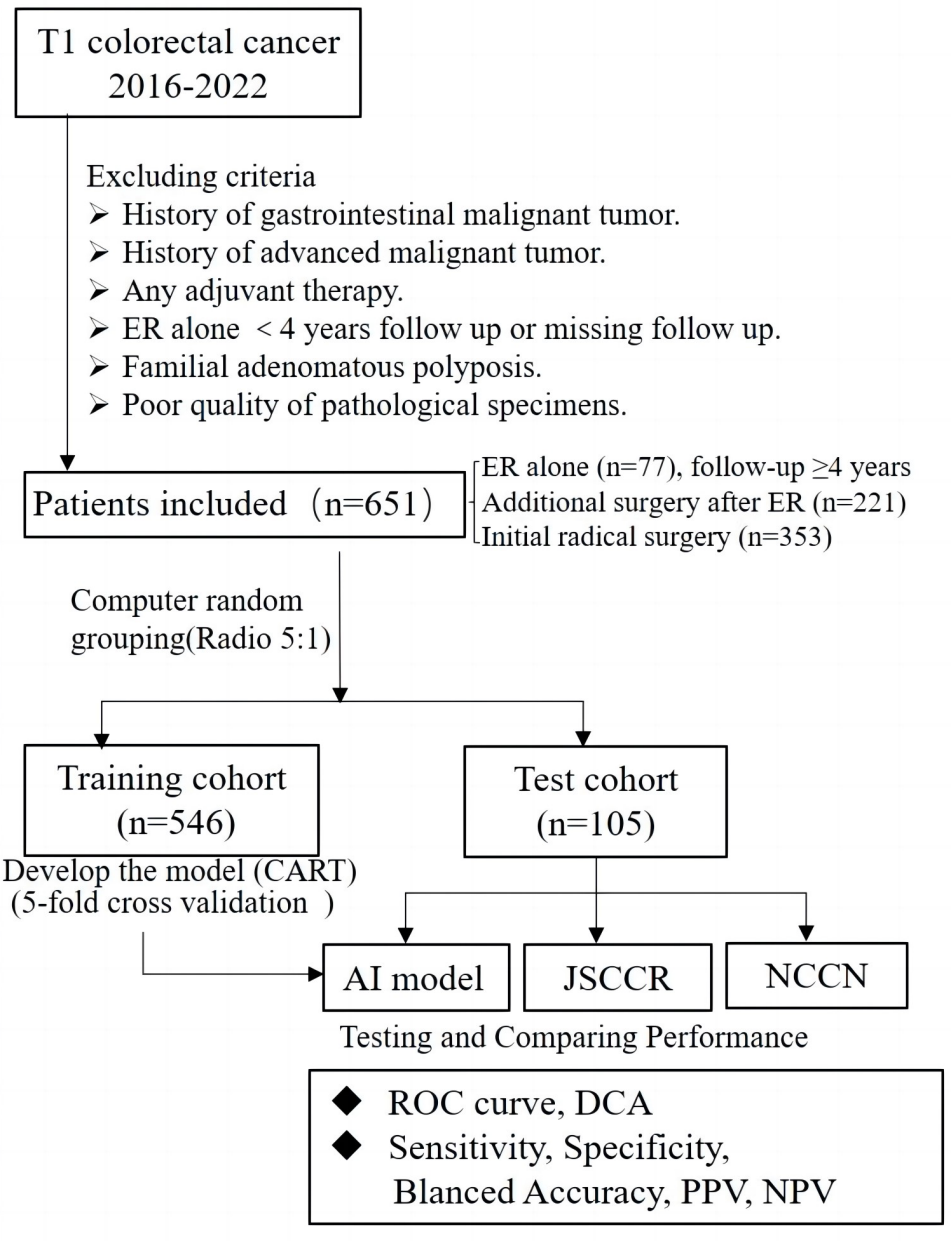
Flowchart of patients included in this study for training and test set, and design of this study.

This retrospective study collected data from patients who had been diagnosed with T1 CRCs and underwent either ER or radical surgery at six large general hospitals in Ningbo, China, from 2016 to 2022. The exclusion criteria were as follows: 1) patients with any history of malignant gastrointestinal tumors; 2) patients with any history of advanced malignant tumors; 3) patients who underwent ER alone and were lost to follow-up or completed less than four years of follow-up after ER; 4) patients who received any adjuvant therapy; 5) patients with familial adenomatous polyposis; and 6) patients that could not be evaluated due to poor quality of pathological specimens. Ultimately, a total of 651 cases were enrolled in this study, including cases of ER alone(77 cases), additional surgery after ER(221 cases), and initial radical surgery(353 cases). Operative specimens were used as the gold standard for the presence of LNM, and patients who underwent ER alone with no evidence of recurrence during the follow-up period (≥4 years) were regarded as negative for LNM. Clinical decisions and management followed the “Guidelines of Chinese Society of Clinical Oncology, Colorectal Cancer” in all six hospitals. Follow-up in these patients was performed according to routine clinical care protocols. The overall scheme of the workflow is illustrated in **Figure 1**.

### Assessment of clinicopathological factors

**Figure 2:**
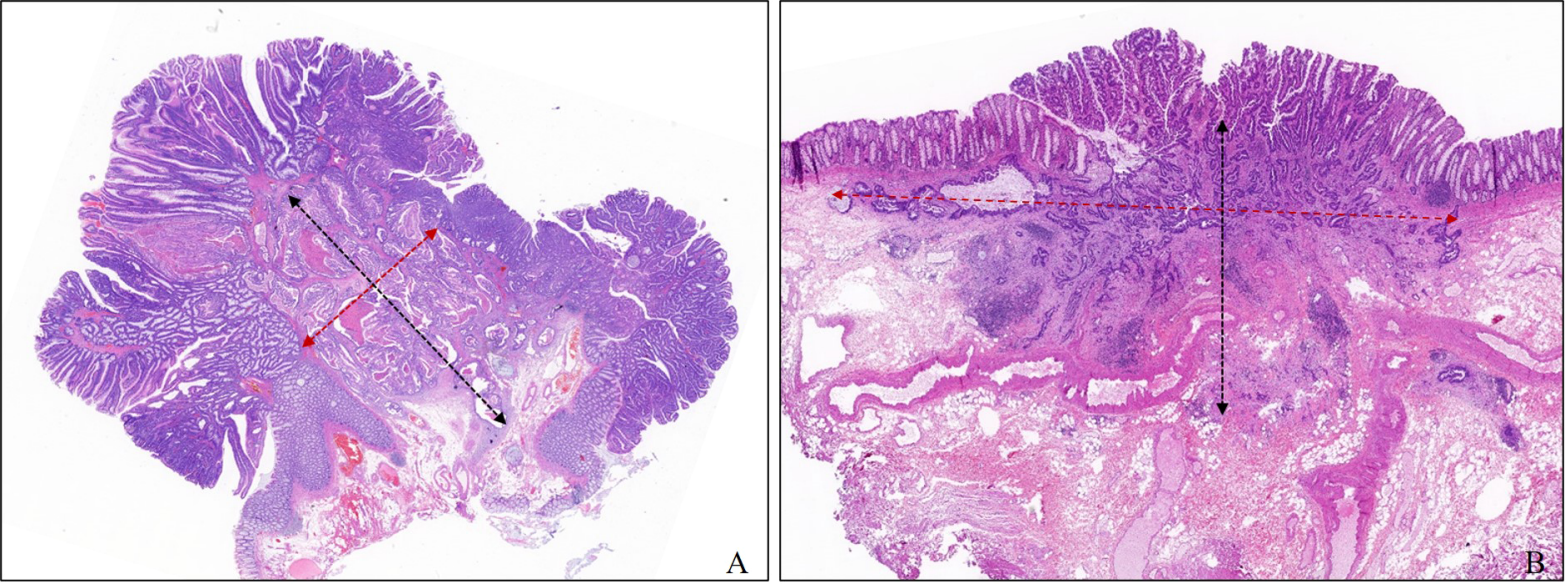
Measurement method of depth and width of submucosal invasion in this study. The width and the depth of submucosal invasion are measured in the widest and deepest parts of the submucosal infiltration, respectively. **(A)** Pedunculated. **(B)** Sessile.

We collected the age, sex, tumor size, location, and morphology and operation records of each patient from the hospital records. Lymph node status was obtained directly from the examination of radical surgical specimens or follow-up visits. All factors used to develop the model were re-evaluated in all slides by one experienced digestive pathologist who conducted a professor consultation when there was disagreement with the original pathological diagnosis. The evaluated histologic factors included histologic differentiation, lymphovascular invasion (LV), tumor budding, poorly differentiated clusters (PDCs), width of submucosal invasion (WSI), depth of submucosal invasion (DSI), and area of submucosal invasion (ASI). WSI was measured at the widest part of the range of submucosal invasion. The method for measuring DSI was as follows regardless of the morphology: when the muscularis mucosae could be identified or estimated, DSI was measured from the lower border of the muscular mucosae; when the muscularis mucosae could not be identified or estimated, DSI was measured from the surface layer of the lesion, but the residual adenoma components on the surface of the lesion were not included. In this study, we defined the product of the DSI and WSI as the area of submucosal invasion (ASI) **Figure 2**. Histologic subtype and grade were evaluated based on the World Health Organization Classification of Tumors. Histological differentiation was divided into well- to moderately differentiated adenocarcinoma, poorly differentiated adenocarcinoma (POR), and well- to moderately differentiated adenocarcinoma with an obvious mucinous adenocarcinoma component (≥30%). Tumor budding was defined as a cancer cell nest consisting of one or fewer than five cells that infiltrated the interstitium at the invasive margin of the cancer, and PDCs were defined as cancer clusters of ≥5 cancer cells infiltrating the stroma and lacking glandular formation. Tumor budding and PDCs were evaluated according to the Japanese Society for Cancer of the Colon and Rectum (JSCCR) guidelines and Ueno’s methods, respectively (13, 14). When LV infiltration and DSI were difficult to judge, cases were evaluated by immunostaining with antibodies against CD34, CD31, D2-40, and Desmin, and cases that were still difficult to assess after immunohistochemistry due to poor specimen quality were excluded.

### Development of the AI model

The cohort of 651 patients was randomly divided into a training set and a test set (ratio 5:1), and the LightGBM algorithm was used to train the 546 patients in the training set to develop a predictive model for LNM. We performed 5-fold cross validation as follows: for each set of parameters, we fitted the model to 4/5 of the data, used 1/5 of the data for internal validation, and then rotated the data through the validation set 1/5 at a time until every data point had been part of the validation set once. The AI model was developed using the following 12 factors: age, sex, location, tumor size, tumor morphology, LV, histologic features, tumor budding, PDCs, WSI, DSI and ASI. Age, tumor size, WSI, DSI, and ASI were recorded as continuous variables. Missing values were imputed using the mice package and filled by predictive mean matching. The permutation feature importance method was selected during the feature selection process. Each feature was randomly arranged, and the model was evaluated on the perturbed data. Finally, the performance difference between the model on the original data and the perturbed data was compared. The ratio of LNM+ and LNM- cases was 59:487 in the training set. We did not perform balanced processing and did not weigh the prediction results of the model. We used LightGBM’s built-in function to obtain the feature importance score, and to more intuitively reflect the importance of each feature, we normalized it. We conducted hyperparameter debugging in the validation set, and based on the experience of AI model developers, we tested the model’s performance on the test set. The threshold was selected in steps of 0.01 starting from 0 for validation, and fine-tuning was performed on the optimal performance interval of the model.

### Assessment of the AI model

To compare the performance of the AI model with the conventional guidelines, the test set was evaluated according to the Japanese Society for Cancer of the Colon and Rectum (JSCCR) and National Comprehensive Cancer Network (NCCN) guidelines. According to the guidelines, patients who have any one of the risk factors in pathological examination are classified as a high-risk group for LNM. **Table 1** shows the criteria for screening high-risk LNM patients in the guidelines. The performance of the AI model was measured by the area under the ROC curve (AUC), sensitivity, specificity, balanced accuracy, positive predictive value (PPV), negative predictive value (NPV), and decision curve analysis (DCA) in the test set and compared with the performance of the JSCCR and NCCN guidelines.

**Table 1:** Indications for additional treatment after endoscopic resection of T1 colorectal cancer.

Guidelines	Vertical margin	LV	High-grade histology	Mucinous adenocarcinoma	Tumor budding	DSI
JSCCR	✓	✓	✓	✓	✓	✓
NCCN	✓	✓	✓			

LV, lymphovascular invasion; DSI, depth of submucosal invasion; JSCCR, Japanese Society for Cancer of the Colon and Rectum; NCCN, National Comprehensive Cancer Network.

### Statistical analysis

The ROC curve and decision curve were plotted using Python (version 3.8). All statistical analyses were performed using R software (version 4.3.0), and p <0.05 was considered statistically significant. Chi-square test and wilcoxon test were used to determine the significance of differences between groups for dichotomous and continuous variables. Confidence intervals (CIs) were calculated using the binom test.

## Results

### Baseline characteristics of the patients

**Figure 3:**
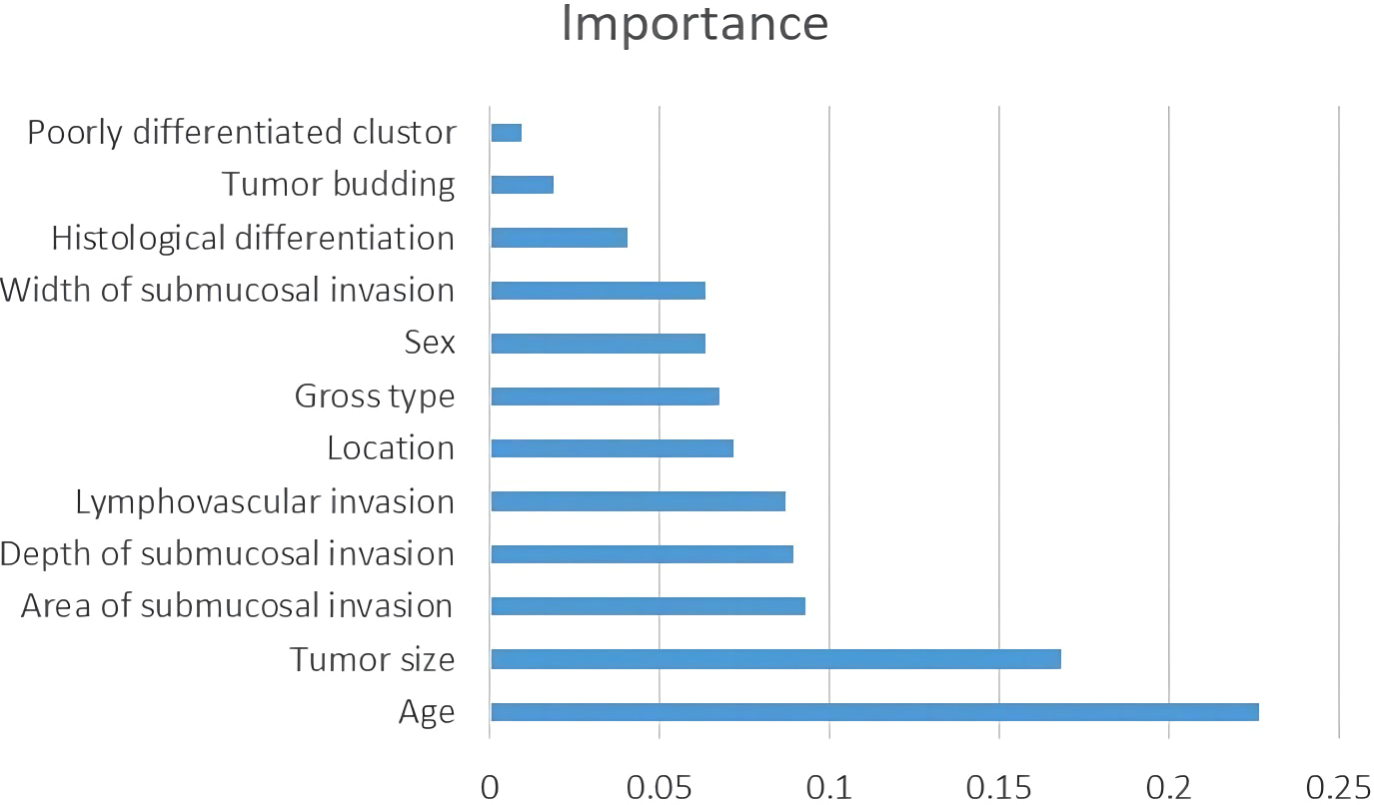
Factor importance of the developed model.

**Table 2** shows the baseline characteristics of the population participating in this study. A total of 651 patients were enrolled in this study, comprising 546 cases (83.7%) as the training cohort and 105 cases (16.3%) as the test set for model development and validation. Among the 221 patients who underwent additional radical surgery after ER, 219 underwent additional radical surgery within one month, and two patients underwent additional radical surgery at 10-12 months of follow-up due to suspected LNM. There was no significant difference in all of the factors and in the rate of LNM in total (10.8% vs. 7.6%, p=0.325) or subgroups (ER alone group 0% vs. 0%, p>0.999; ER+surgery group 8.6% vs. 2.8%, p=0.242; initial surgery group 14.7% vs. 11.4%, p=0.508) between the training and testing sets. The average number of lymph nodes per patient in the training and test sets was 11 ± 5 (median, 10) and 12 ± 5 (median, 12), respectively. **Figure 3** shows the importance of each factor used in the development of the AI model. Age and tumor size had the most important effects on the ability to predict LNM among the examined factors.

**Table 2:** Baseline characteristics of the training and test sets.

		Total(651) n(%)	Training set(546) n(%)	Test set(105) n%)	P value
Age(y) [median, IQR]		65(57-71)	64(56-71)	66(57-72)	0.438
Sex (Male)		402(61.7)	335(61.3)	67(63.8)	0.716
Tumor size(mm) [median, IQR]		20(15-28)	20(15-28)	20(15-30)	0.653
Location	Ileocecum	4(0.6)	4(0.7)	0(0)	0.843
	Ascending colon	61(9.3)	45(8.2)	16(15.2)	0.038
	Transverse colon	23(3.5)	22(4.0)	1(0.9)	0.202
	Descending colon	22(3.3)	20(3.6)	2(1.9)	0.536
	Sigmoid colon	189(29.0)	162(29.6)	27(25.7)	0.483
	Rectum	352(54.0)	293(53.6)	59(56.1)	0.712
Morphology	Pedunculated	243(37.3)	207(37.9)	36(34.3)	0.553
	Sessile	408(62.7)	339(62.1)	69(65.7)	0.553
Histologic features	Low-grade	582(89.4)	484(88.6)	98(93.3)	0.209
	High-grade	54(8.2)	49(8.9)	5(4.7)	0.215
	MUC components	15(2.3)	13(2.3)	2(1.9)	>0.999
LV	Positive	127(19.5)	107(19.5)	20(19.0)	>0.999
Tumor budding	G2-G3	26(3.9)	19(3.5)	7(6.7)	0.209
PDC	G2-G3	24(3.6)	17(3.1)	7(6.7)	0.137
WSI (mm) [median, IQR]		9.0(6.0-13.0)	8.5(6.0-12.5)	9.0(6.1-13.0)	0.862
DSI (mm) [median, IQR]		4.0(2.0-5.5)	4.0(2.4-6.0)	4.0(2.5-6.5)	0.874
ASI (mm) [median, IQR]		31.5(11.2-60.0)	35.1(14.0-79.0)	37.5(18.3-72.0)	0.815
Treatment	ER alone	77(11.8)	68(12.4)	9(8.5)	0.335
	ER+Surgery	221(33.9)	186(34.0)	35(33.3)	0.974
	Initial Surgery	353(54.2)	292(53.4)	61(58.0)	0.446
LNM	Positive	67(10.2)	59(10.8)	8(7.6)	0.419

LV, lymphovascular invasion; PDC, poorly differentiated cluster; WSI, width of submucosal invasion; DSI, depth of submucosal invasion; ASI, area of submucosal invasion; LNM, lymph node metastasis; ER, endoscopic resection; Chi-square test and wilcoxon test were used to determine the significance of differences between groups for dichotomous and continuous variables.

### Performance of the AI model

**Figure 4:**
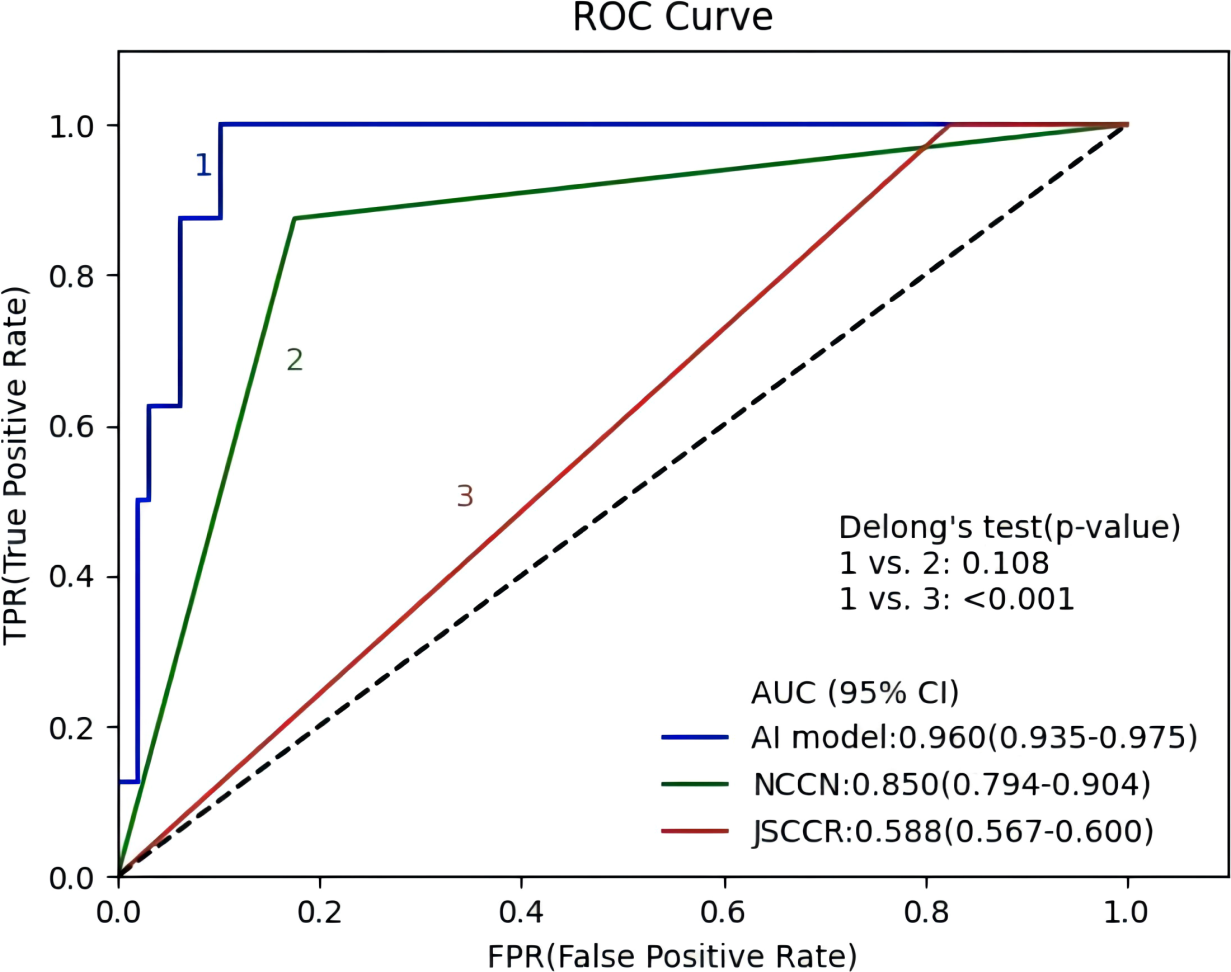
Receiver operating characteristic curves of the artificial intelligence model, Japanese Society for Cancer of the Colon and Rectum (JSCCR), and National Comprehensive Cancer Network (NCCN) guidelines.

**Figure 5:**
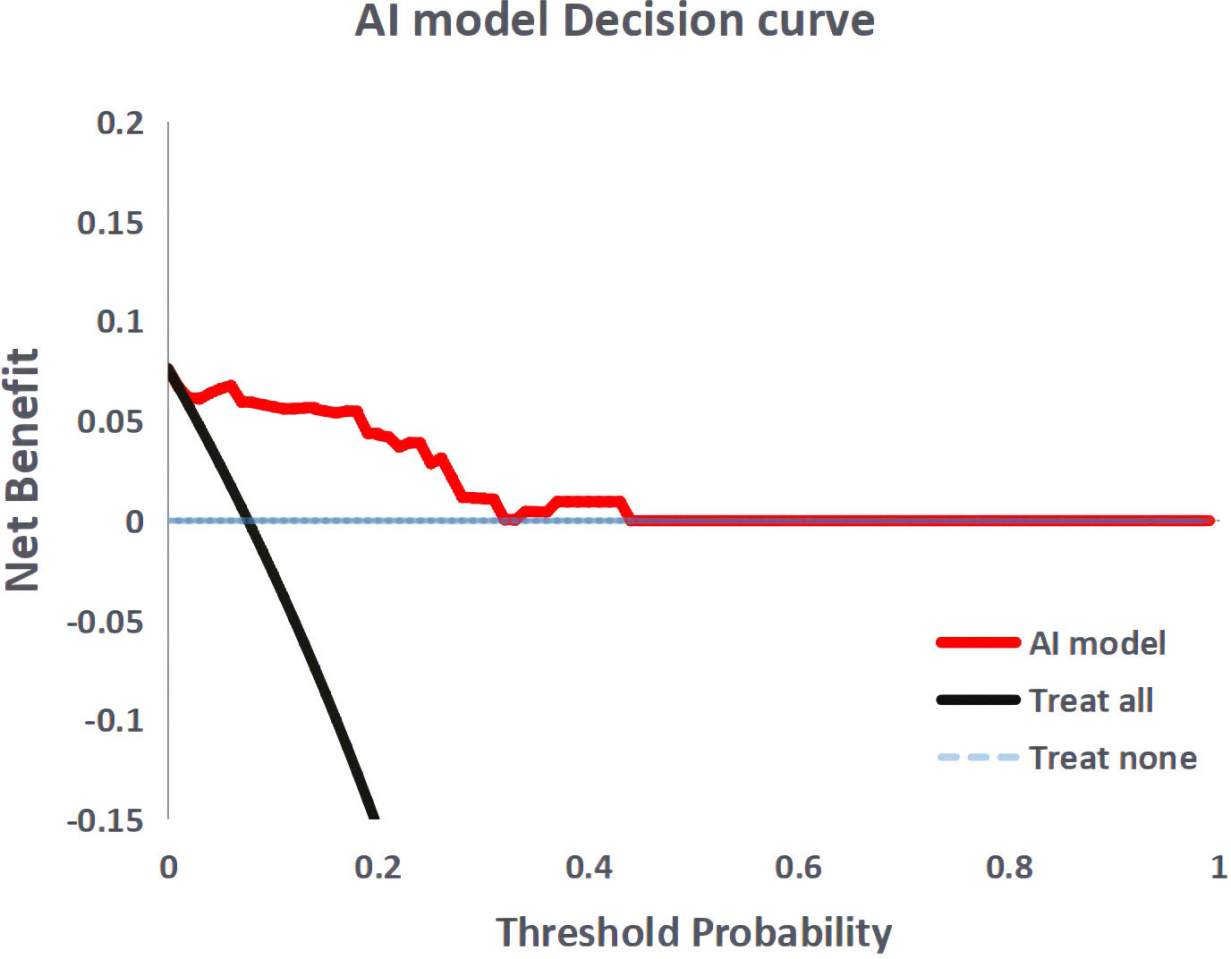
Decision curve analysis for the artificial intelligence model. The black dashed line the assumption that no patient with lymph node metastasis, and the solid line represents all patients with lymph node metastasis.

**Figure 4** shows the ROC curves of the AI model and guidelines on the test set. As quantified by the AUC, the AI model had better performance (AUC=0.960) than the JSCCR guidelines (AUC=0.588) or the NCCN guidelines (AUC=0.850). DCA showed a positive net benefit from the use of the AI model on the test set **Figure 5**. **Tables 3**, **4** show the predictive performance of the AI model and guidelines. On the test set, the AI model, JSCCR, and NCCN guidelines predicted that 22 cases, 88 cases, and 24 cases, respectively, were positive for LNM; among them, 8 cases, 8 cases, and 7 cases were truly positive, respectively. When the threshold was set to 0.06, the sensitivity and specificity of the AI model were 100% (95% CI, 63% to 100%) and 85.8% (95% CI, 76% to 91%), respectively. According to our AI model, 79.0% of patients could avoid additional surgery. The specificity (85.8% vs. 17.5%, *p*<0.001), balanced accuracy (92.9% vs. 58.7%, *p*=0.001), and PPV (36.3% vs. 9.0%, *p*=0.001) of the AI model were significantly better than those of the JSCCR guidelines. According to the AI model, the proportion of patients deemed to be at high risk for LNM was reduced from 83.8% (JSCCR) to 20.9%. The specificity (85.8% vs. 82.4%, p=0.557) and sensitivity (100% vs. 87.5%, p=0.301) of the AI model were higher than those of the NCCN guidelines, but according to the NCCN guidelines, 1.2% of patients would have a missed diagnosis.

**Table 3:** Predicted results of the artificial intelligence model, JSCCR, and NCCN in the test set.

	Prediction	Test set (n=105)	Actual	
		n (%)	LNM (-), n (%)	LNM (+), n (%)
AI model	LNM(-), Low-risk	83(79.0)	83(100)	0(0)
	LNM(+), High-risk	22(20.9)	14(63.6)	8(36.3)
JSCCR	LNM(-), Low-risk*	17(16.2)	17(100)	0(0)
	LNM(+), High-risk*	88(83.8)	80(90.9)	8(9.1)
NCCN	LNM(-), Low-risk*	81(77.2)	80(98.7)	1(1.2)
	LNM(+), High-risk*	24(22.8)	17(70.8)	7(29.2)

AI, Artificial intelligence; JSCCR, Japanese Society for Cancer of the Colon and Rectum; NCCN, National Comprehensive Cancer Network; LNM, lymph node metastasis; *High/Low risk: With or without any risk factors.

**Table 4:** Predictive performance of the artificial intelligence model, JSCCR, and NCCN guidelines for lymph node metastasis in patients with T1 colorectal cancer.

	AI model (95%CI)	JSCCR(95%CI)	NCCN(95%CI)	*P* value*	*P* value**
Sensitivity	100 (63 to 100)	100 (59 to 100)	87.5(46 to 99)	>0.999	0.301
Specificity	85.8 (76 to 91)	17.5 (10 to 26)	82.4(73 to 89)	<0.001	0.557
Balanced Accuracy	92.9 (74 to 95)	58.7 (35 to 63)	84.9(59 to 94)	0.001	0.841
PPV	36.3(17 to 59)	9.0 (4 to 17)	29.1(13 to 51)	0.001	0.603
NPV	100(95 to 100)	100 (77 to 100)	98.7(92 to 99)	>0.999	0.310

AI model, artificial intelligence model; JSCCR, Japanese society for cancer of the colon and rectum; NCCN, national comprehensive cancer network; CI, confidence interval; PPV, positive predictive value; NPV, negative predictive value. P value*,AI model vs JSCCR; P value**, AI model vs NCCN.

## Discussion

Effectively screening for patients who are at risk for LNM after ER is the key to ensuring a good prognosis and reducing overtreatment. In this study, we developed a prediction model for LNM in patients with T1 CRC using a decision tree algorithm; the model achieved excellent predictive performance, outperforming the JSCCR and NCCN guidelines.

At present, LV, high-grade histology, DSI, and tumor budding are recognized as risk factors for LNM (1–3). However, each factor has a different weight on the correlation with LNM, and their different combinations have different correlations with LNM. For example, LV has been shown to be an independent risk factor for LNM in most studies, while DSI often shows a very weak correlation (8, 15–17). Different patients often have different types and combinations of risk factors; therefore, these patients have different risks of LNM. In addition, scholars have reported in some studies that the gross type (18, 19), location (20), and size of the tumor (21) and the age (21) and sex (11, 22) of the patient are also related to LNM; however, guidelines are unable to comprehensively reflect the role of these potential influencing factors. In a few studies, scholars have attempted to grade the risk of LNM based on a combination of different risk factors (8–11). Miyachi et al. (11) attempted to use 5 factors to grade LNM risk, and 64% of patients were classified in the high to ultrahigh group. Our previous study with a small cohort showed that in patients with T1 CRCs treated by ER, the risk of LNM can be divided into low, moderate, and high grades based on different combinations of LV, histological differentiation, and other risk factors; the rates of LNM in these groups were 0.8%, 25.0-28.8%, and 66.6%, respectively (8).

Recently, predictive models using AI technology have gradually been developed to predict medical outcomes, and those models have outperformed the guidelines (6, 7, 23–25). The performance of the predictive model largely depends on the accuracy of the variables and the selection of algorithms during model development. To ensure the accuracy of each variable, we re-evaluated each slide microscopically. We developed the model based on 12 variables using a decision tree algorithm, and its performance was superior to that of the guidelines. In this study, in addition to widely recognized factors such as LV, high-grade histology, tumor budding, and DSI, we included patient age, sex, and tumor location as variables in the model development, as the potential correlation of these factors with LNM remains unclear. LV invasion is a requirement for LNM in T1 colorectal cancer. Therefore, we believe that the ASI correlates with LNM because the larger the ASI is, the greater the opportunity for cancer cells to contact the LV. Of course, whether it will invade the vasculature also depends on the invasiveness of the cancer cells. Therefore, we added the ASI to the model development as a variable. Although the AI model showed high performance, how each factor influences the outcome is difficult to speculate. Hence, we calculated and digitized the importance of each factor used in the AI model. The results showed that age and tumor size were the most important factors for prediction. For us, this result was unexpected because multivariate analysis in most studies shows that these factors are not independent risk factors for LNM in T1 CRCs patients who are treated by ER. Ahn et al. (21) developed prediction models for LNM in T1 CRC using five types of machine learning algorithms, and their results also showed that patient age, sex, and tumor size are important factors in predicting LNM.

Our AI model (AUC=0.960) showed better predictive performance on the test set than the JSCCR (AUC=0.588) or NCCN (AUC=0.850) guidelines. Although the sensitivity of the JSCCR guidelines is 100%, 83.8% of patients are classified in the high-risk group. The specificity of the AI model was significantly higher than that of JSCCR (85.8% vs. 17.5%, p<0.001), and according to the AI model, the proportion of high-risk patients was reduced from 83.8% to 20.9%. Although the specificity of NCCN also reaches 82.4%, 1.2% of patients will be missed in the diagnosis according to NCCN guidelines.

Predictive models for LNM in T1 CRCs have been developed in a small number of studies using machine learning algorithms, and all of them showed better predictive performance than the guidelines. It was reported that an AI model using the least absolute shrinkage and selection operator (LASSO) method (AUC=0.76-0.83) outperformed the American Society for Gastrointestinal Endoscopy (ASGE)/European Society for Gastrointestinal Endoscopy (ESGE) guidelines (AUC=0.67) and the JSCCR (AUC=0.518-0.65) guidelines (6, 25). Ichimasa et al. (7) developed an AI model using a support vector machine (SVM), and the model achieved significantly higher specificity (66% for the model vs. 0% - 44% for the guidelines) and accuracy (69% for the model vs. 9% - 49% for the guidelines) than the NCCN, European Society for Medical Oncology (ESMO), and JSCCR guidelines. The ANN model (AUC=0.83) developed by Kudo et al. (23) outperformed the US (AUC=0.73) and Japanese (AUC=0.57) guidelines. In this study, we used the decision tree algorithm (AUC=0.960), which showed better performance than the other above models. Ahn et al. (21) compared the performance of T1 CRC LNM prediction models developed using five types of machine learning algorithms and found that the random forest (AUC=0.991) and CART (AUC=0.944) models had better performance than other models; both models were similar to ours in performance.

This study has several limitations. First, approximately 10% of patients in this study did not receive additional surgery because there were no risk factors detected after ER, and their LNM status was assessed only by endoscopic and imaging examinations, although most of these patients were followed up for more than 5 years. Second, although our AI model exhibits excellent performance in the test set, sufficient external validation is required to further confirm its performance before it can be applied in clinical practice.

In conclusion, our AI model has better performance than conventional guidelines for predicting LNM in T1 CRCs and can effectively reduce unnecessary additional surgery. We propose that AI prediction models be routinely applied to inform treatment decisions in patients who have undergone ER of T1 CRCs.

## Data availability statement

The raw data supporting the conclusions of this article will be made available by the authors, without undue reservation.

## Ethics statement

The studies involving humans were approved by The data and experiments reported herein were performed in accordance with the Declaration of Helsinki principles and the Ethics Committee of the Ningbo Diagnostic Pathology Center. The studies were conducted in accordance with the local legislation and institutional requirements. The human samples used in this study were acquired from primarily isolated as part of your previous study for which ethical approval was obtained. Written informed consent for participation was not required from the participants or the participants’ legal guardians/next of kin in accordance with the national legislation and institutional requirements.

## Author contributions

ZHP and RG conceived and designed this study. ZHP, and LL collected and analyzed the data. ZHP drafted and revised this manuscript. All authors contributed to the article and approved the submitted version.
